# Symptomatic SARS-CoV-2 infections after full schedule BNT162b2 vaccination in seropositive healthcare workers: a case series from a single institution

**DOI:** 10.1080/22221751.2021.1942230

**Published:** 2021-06-22

**Authors:** Andreina Baj, Federica Novazzi, Angelo Genoni, Francesca Drago Ferrante, Stefano Taborelli, Beatrice Pini, Michele Partenope, Marilena Valli, Daniela Dalla Gasperina, Riccardo Capuano, Martina Prestia, Pietro Giorgio Spezia, Lorenzo Azzi, Daniele Focosi, Fabrizio Maggi

**Affiliations:** a Department of Medicine and Surgery, University of Insubria, Varese, Italy; b Laboratory of Microbiology, ASST dei Sette Laghi, Varese, Italy; c Department of Biotechnology and Life Sciences, University of Insubria, Varese, Italy; d Unit of Occupational Medicine, ASST dei Sette Laghi, Varese, Italy; e Clinical Pathology Unit, St. Anna Hospital, Como, Italy; f Department of Translational Research, University of Pisa, Pisa, Italy; g Unit of Oral Medicine and Pathology, ASST dei Sette Laghi, Varese, Italy; h North-Western Tuscany Blood Bank, Pisa University Hospital, Pisa, Italy

**Keywords:** COVID19, SARS-CoV-2, vaccine, BNT162b2, variants of interest

## Abstract

We report 11 cases of SARS-CoV-2 infection in healthcare workers (HCW) *naïve* for COVID-19 and seropositive after the second dose of the BNT162b2 mRNA vaccine. Based on voluntary-based surveillance, they tested positive for different strains of SARS-CoV-2, as Spike gene sequencing showed. Five of them reported mild symptoms. Given the risk for SARS-CoV-2 introduction from asymptomatic vaccinees, this case series suggests the need to continue nasopharyngeal screening programmes.

Dear Editor, the immune response to the COVID-19 vaccine is typically established by measuring anti-SARS-CoV-2 Spike or anti-RBD IgG in serum, even if no information about IgA levels in respiratory secretions or saliva is available. Playing a pivotal role to prevent SARS-CoV-2 infection and transmission, this has obvious implications for the final reach of herd immunity.

In Italy and many European countries, vaccination campaigns have been started in December 2020 with the BNT162b2 mRNA vaccine Comirnaty® (Pfizer/BioNtech), prioritizing healthcare workers (HCW). The susceptibility of vaccinated HCW to active, albeit asymptomatic, infection is of high interest given the risk of transmitting the virus to frail hospitalized patients. Additionally, the asymptomatic status in the vaccinated HCW could delay recognition of the index case, favouring nosocomial outbreaks.

We report here 11 HCW (mean age: 50±10 years, 3 male and 8 female) who tested positive for SARS-CoV-2 (m2000, Abbott Molecular, Des Plaines, IL), with a mean cycle threshold (Ct) of 19 (range Ct: 6–26) in nasopharyngeal swabs (NPS) after a variable period from the second dose of vaccine (mean days: 43±10). They were part of voluntary-based surveillance, according to the guidelines of the Italian National Institute of Health and Ministry of Health and Local Ethical Committee (protocol n° 165/2020). All of them were *naïve* to COVID-19 and had successfully mounted anti-Spike serum IgG (LIAISON® SARS-CoV-2 S1/S2 IgG, DiaSorin, Saluggia, Italy) after full schedule vaccination, with a mean antibody titer of 214±99 arbitrary units (AU) for ml. While most cases were asymptomatic for the entire time between the first positive and the first negative NPS, 5 reported fever and arthralgia. Among them, cases #9 and #11 are notable because of positivity in rectal swab. Given the ECDC and Italian Ministry of Health recommendation to sequence SARS-CoV-2 isolated from vaccinees to detect immune-escaping variants of concern [1], we sequenced the entire Spike gene in all 11 cases. Sequences were deposited on GenBank and characterized using NextClade (https://clades.nextstrain.org/), showing the synchronous circulation of different SARS-CoV-2 strains in the Lombardy region. Table 1 summarized the laboratory findings in these cases.

**Table 1.** Summary of laboratory findings in the 11 vaccinated HCW.

#	Gender/ Age (yrs)	From 2nd dose to positive NPS (days)	rtPCR Ct	Anti-S1/S2 IgG (AU/ml)	SARS CoV-2 RNA in rectal swab/saliva	Symptoms	From 1st positive to 1st negative NPS (days)	Spike protein (aa substitutions)	Variant	GenBank ID
1	F/61	30	24	174	NA	None	1	R346S, L452R, N501Y, A570D D614G, Q677H P681H, A899S del69-70, del144	UK (20I/501Y.V1 or B.1.1.7)	MW826653
2	M/37	34	26	209	NA	None	1	N501Y, A570D	UK (Y380Y+ V433V)	MW826670
3	F/61	52	20	205	NA	fever, arthralgia, rhinitis	NA	L452R, N501Y, A570D	UK + L452R	MW834428
4	M/60	23	25	294	NA	None	13	N501Y, A570D D614G, Q677H P681H, A899S S982A, del69-70, del144	UK (20I/501Y.V1 or B.1.1.7)	MW826840
5	F/55	57	22	123	NA	fever, arthralgia, rhinitis	NA	N501Y, A570D	UK (20I/501Y.V1 or B.1.1.7)	MW834320
6	F/50	51	16	131	NA	fever, arthralgia, rhinitis	15	N501Y, A570D, D614G, P681H, T716I, S982A, D1118H, del69-70, del144	UK (20I/501Y.V1 or B.1.1.7)	MW827173
7	F/28	44	NA	397	Negative/ Negative	rhinitis, anosmia, cough	12	None	–	MW815132
8	F/48	47	16	185	NA	None	2	None	–	MW827227
9	F/56	48	6	110	Positive (Ct 27)/ Positive (Ct 15)	fever, arthralgia, rhinitis	15	N501Y, A570D	UK (20I/501Y.V1 or B.1.1.7)	MW827231
10	M/44	48	NA	158	NA	none	4	N501Y, A570D	UK (20I/501Y.V1 or B.1.1.7)	MW827585
11	F/55	48	24	378	Positive (Ct 24)/ Positive (Ct 33)	fever, arthralgia, rhinitis	4	N501Y, A570D	UK (20I/501Y.V1 or B.1.1.7)	MW827588

Nevertheless the documented efficacy of BNT162b2 mRNA vaccine (7), at least one case of asymptomatic infection in a vaccinated HCW has been recently reported [2], but since the biological samples were limited to NPS, it could not be concluded whether the infection had been contained on the nasopharynx or not. Our series is the first to demonstrate that in fully vaccinated HCW, SARS-CoV-2 is not only able to colonize the nasopharynx, but also to infect cells in distant tissues and give clinical symptoms. No case of nosocomial transmission from the HCW described in this series to inpatients has been documented to date, even if the low Ct found is a suggestion of a viable and transmissible virus.

While we cannot conclude whether the detected viral RNA was immunocomplexed or not, it is well known that sterilizing immunity against respiratory viruses largely depends on neutralizing IgA levels in secretions. Unfortunately, the intramuscular route of the currently approved vaccines only induces low-level IgA in secretions in a minority of recipients [3–5].
